# Proteomics-driven screening of artemisinin-based combination ratios and mechanistic insights into *Plasmodium berghei* infection in mice

**DOI:** 10.3389/fimmu.2025.1716096

**Published:** 2025-12-05

**Authors:** Liyu Hao, Jianhui Sun, Jianliang Li, Zongyuan Li, Zeyue Yu, Hanhui Huang, Guimin Liu, Zhenru Shen, Hairu Huo, Qili Yuan, Hongmei Li, Luqi Huang

**Affiliations:** 1 School of Traditional Chinese Medicine, Shenyang Pharmaceutical University, Shenyang, China; 2 Institute of Chinese Materia Medica, China Academy of Chinese Medical Sciences, Beijing, China; 3 Institute of Traditional Chinese Medicine Health Industry, China Academy of Chinese Medical Sciences, Nanchang, China; 4 China Academy of Chinese Medical Sciences, Beijing, China

**Keywords:** artemisinin, artemisinin-based combination therapy, drug resistance reversal, Fcγ receptor signaling, Malaria

## Abstract

**Introduction:**

Malaria, a life-threatening mosquito-borne disease caused by *Plasmodium falciparum*, poses a substantial health burden on tropical and subtropical regions. Artemisinin, a sesquiterpene lactone isolated from *Artemisia annua* L., and its derivatives were initially used as monotherapies for malaria treatment. However, limitations such as short pharmacokinetic half-life and emerging drug resistance have driven the widespread adoption of artemisinin-based combination therapies (ACTs) as first-line interventions. *A. annua* contains other bioactive compounds such as arteannuin B, artemisinic acid, and scopoletin that exhibit distinct pharmacological properties. In this study, we aimed to devise a new strategy for treatment of malaria to overcome artemisinin resistance in *Plasmodium* species.

**Methods:**

We systematically screened antimalarial compound ratios using murine malaria models and optimized a formula comprising arteannuin B, artemisinic acid, and scopoletin. Through integrated proteomic profiling and western blot validation, we elucidated the immunomodulatory mechanisms underlying the antimalarial efficacy of this combination.

**Results:**

Specifically, the formula strengthened host defense by modulating phagocytic activity in splenic macrophages, dendritic cells, and natural killer cells via Fcγ receptor-mediated pathways.

**Discussion:**

These findings provide mechanistic insights into artemisinin-associated immune potentiation. Moreover, we have proposed a novel ACT strategy targeting host-parasite interactions, offering a promising approach to circumvent emerging artemisinin resistance in *Plasmodium* species.

## 1 Introduction

Malaria is an insect-borne infectious disease caused by *Plasmodium falciparum*, which is transmitted to humans who are bitten by *Anopheles* mosquitoes infected with *P. falciparum* or imported into the blood of people carrying *P. falciparum* (1). Malaria is distributed in more than 90 countries worldwide, including the tropics and subtropics. Nearly half of the world’s population is at risk of contracting malaria, and its incidence and mortality rates are the highest among insect-borne diseases (2). The “World Malaria Report 2023” (1)released by WHO shows that approximately 249 million cases of malaria were reported worldwide in 2022, leading to approximately 608,000 deaths.

The emergence of antimalarial-resistant *P. falciparum* strains has significantly compromised malaria management strategies in endemic regions worldwide (3). In particular, the widespread prevalence of multidrug-resistant *P. falciparum* has limited the availability of cost-effective therapeutic options in resource-constrained settings (4). This therapeutic challenge is further compounded by the concurrent development of insecticide resistance in *Anopheles* vector populations. This has created a dual-resistance paradigm that exacerbates the challenges associated with malaria control in the affected tropical and subtropical regions (5, 6).

Artemisinin, a sesquiterpene lactone extracted from *Artemisia annua* L., and its derivatives were originally used as a monotherapy to treat malaria. However, the short half-lives of artemisinin drugs and concerns regarding drug resistance have prompted the introduction and implementation of ACTs (7–9).

As early as 2700 BC (10), during the Eastern Jin Dynasty, Ge Hong first mentioned in his handbook “Elbow Reserve Emergency Prescription” that *A. annua* had a therapeutic effect on malaria, and Li Shizhen of the Ming Dynasty also mentioned it in the “Compendium of Materia Medica.” The ancient herbal books in China indicate that *A. annua* can be used to treat deficiencies, heat-related illnesses, malaria, and jaundice, alleviate bone steaming, and relieve summer heat. In addition to artemisinin, other compounds present in *A. annua*, such as arteannuin B (11), artemisinic acid, and scopoletin, also exhibit pharmacological activities (11). Previously, Tu Youyou et al. observed antimalarial activity using a combination of artemisinin with arteannuin B, artemisinic acid, and scopoletin (12).

**Figure 1:**
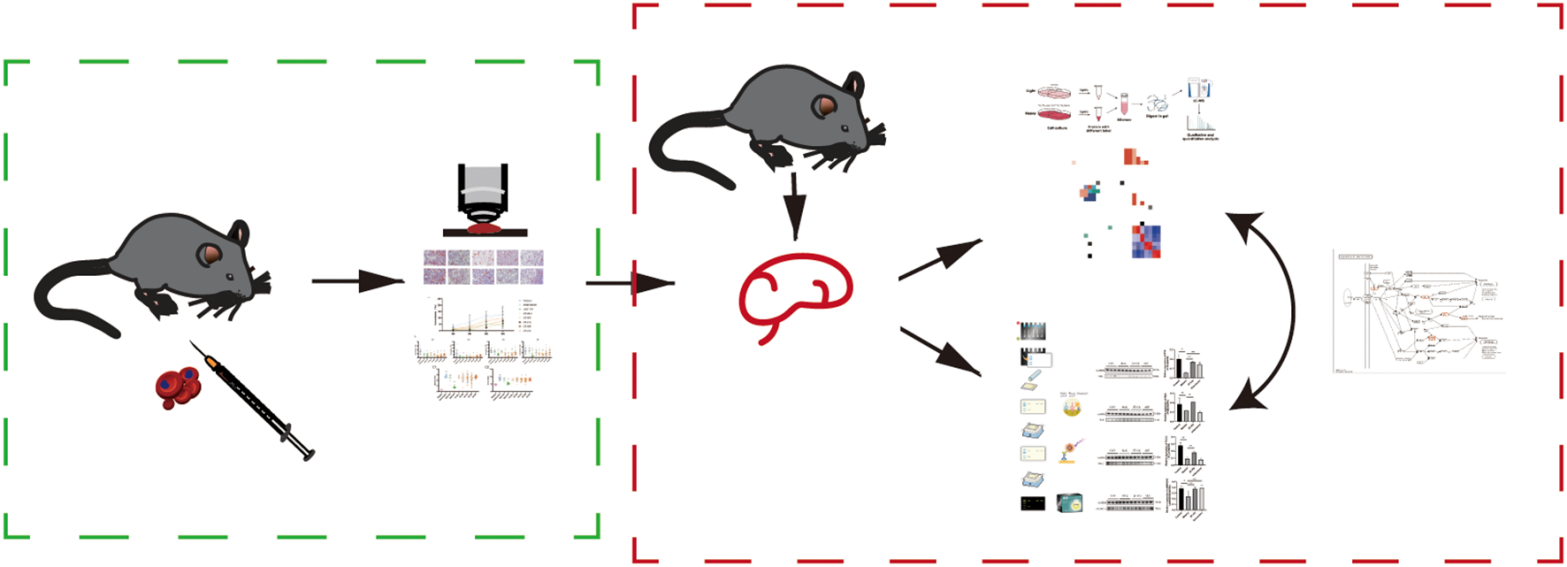
Brief flowchart of overall experimental design.

In the present study, we aimed to devise a new strategy for treatment of malaria to overcome artemisinin resistance in *Plasmodium* species. An artemisinin-resistant strain of *P. berghei* was used to construct a murine malaria model. To screen for a compound with good antimalarial potential, proteomic analysis of the spleen tissue of the host mouse was carried out to explore the mechanism of action of the series of compounds, comprising artemisinin, arteannuin B, artemisinic acid, and scopoletin, from the perspective of host immune regulation. While proposing a novel ACT strategy, this study provides an in-depth analysis of the mechanism of artemisinin-based combination therapies from the host perspective, offering insights for the development of new drugs against drug-resistant *Plasmodium falciparum*. A flowchart of the experimental procedure is shown in **Figure 1**.

## 2 Materials and methods

### 2.1 Ethics statement

All animal procedures were performed in accordance with the Regulations of Experimental Animal Administration (Order No. 2; approved by the State Council in 1988 and third revision in 2017) issued by the State Committee of Science and Technology of the People’s Republic of China. This study was approved by the Institutional Animal Care and Use Committee (IACUC) of the Institute of Chinese Materia Medica, China Academy of Chinese Medical Sciences (Beijing, China) under code number 2023B198.

### 2.2 Experimental animals and parasites

C57BL/6J mice (specific pathogen-free grade; body weight, 18–22 g; equal male and female distribution) were purchased from Beijing Vital River Laboratory Animal Technology Co., Ltd. (Beijing, China; license no.: SCXK<jing> 2021-0006) and housed in the Animal Center (Beijing, China; license no.: SYXK<Jing> 2019-0003) at 20–26°C and 40–70% humidity under standard 12:12 h light–dark cycles. The mice were acclimatized for three days with free access to standard chow (Beijing Keao Xieli Feed Co., Ltd., Beijing, China; cat. no.: 21063213) and sterile water.

The artemisinin-resistant strain *P. berghei* K173 (*Pb* K173) was introduced by Dr. Peters at the London School of Tropical Medicine and Hygiene, UK. The strain was preserved by blood transmission or freezing at the Artemisinin Center of the China Academy of Traditional Chinese Medicine.

### 2.3 Grouping and dosing

Mice were categorized into the following groups according to body weight: control, malaria, artemisinin (ART), and positive control (artemisinin + piperaquine phosphate [ART + PP]) groups and groups with different compositions of antimalarial compounds (ZF-HLJ, ZF-HN, ZF-CQ, ZF-HB, and ZF-GX). Each group consisted of 10 mice (five males and five females).

The positive control drug comprised artemisinin (cat. no.: 20051242) and piperaquine phosphate (cat. no.: 2104062), both procured from Beijing Bethealth People Biomedical Technology Co.,Ltd. The clinical equivalent doses of artemisinin and piperaquine phosphate selected for this experiment were 16.3 and 97.5 mg/kg, respectively.

The monomers of artemisinin, arteannuin B (cat. no.: 20120101), artemisinic acid (cat. no.: 20102241), and scopoletin (cat. no.: 21040621) were procured from Beijing Bethealth People Biomedical Technology Co.,Ltd. The proportions of the four substances in different groups are shown in **Table 1**.

**Table 1:** Composition of four -components in different groups.

Group	Artemisinin (%)	Arteannuin B (%)	Artemisinic acid (%)	Scopoletin (%)
ZF-HLJ	23.8	62.5	10.0	3.7
ZF-HN	58.8	31.6	7.0	2.6
ZF-CQ	81.0	15.7	2.0	1.3
ZF-HB	87.5	8.5	2.2	1.8
ZF-GX	88.6	4.9	3.4	3.0

The proportional relationships of artemisinin, artemisinin B, artemisinic acid, and scopoletin among the antimalarial compounds were designed to simulate the natural ratios of these four components in *Artemisia annua* L. from different geographical regions.

The certificate of analysis for all compound monomers were provided in the **Supplementary Material**-certificate of analysis.

### 2.4 Inoculation of parasite

The artemisinin-resistant *Pb* K173 strain that was frozen in liquid nitrogen was taken out and placed in a water bath at 37°C. After thawing, the strain was immediately injected intraperitoneally into C57BL/6J mice at a dose of 0.2 ml per animal. Mice exhibiting a parasitemia level of approximately 20% were used as donors of the *Pb* K173 strain during the experiment. The jugular veins of the donor mice were severed after euthanasia to collect blood (13). After the donor mice had been slaughtered by decapitation, blood was extracted via cardiac puncture and transferred into a heparinized vacutainer tube containing 0.5% trisodium citrate. Subsequently, the blood was diluted with physiological saline solution (0.9%) according to the donor mouse’s parasitemia level and number of red blood cells (RBCs) in the normal mice; overall, 1 ml of blood contained 5 × 10^7^ infected RBCs (14). Afterward, 0.2 ml of this diluted blood, which contained 1 × 10^7^ RBCs infected with *P. berghei* (iRBCs) was administered intraperitoneally to each animal.

### 2.5 Evaluation of antimalarial activity against mouse model

#### 2.5.1 Four-day suppressive test

After the acclimatization period, mice in the other groups were intraperitoneally injected with 0.2 ml of iRBCs diluted with physiological saline, except the control group, which comprised the mice on day 0 of vaccination (D0). Mice in the control group were intraperitoneally injected with 0.2 ml of physiological saline. The Peters four-day suppressive test was conducted; on D0–D3, iRBCs were administered continuously for four days, whereas the control and malaria groups were administered the same amount of purified water (20 ml kg^-1^) daily.

Blood was collected from the tail tips of mice on D2, D4, D6, and D8 and placed on glass slides to make blood films. After the blood films were air-dried, they were fixed with methanol and stored until completely air-dried. After dilution with Giemsa (15) (cat. no.: BCCG6252; Sigma, USA) for 20 min, the excess dye was washed, and the surface water was quickly blotted with filter paper and dried. The parasite count was ascertained using an oil immersion objective with 100× magnification under a microscope (14, 16). The percentage parasitemia was calculated using the following formula:


$$\begin{array}{c} \mathrm{Percentage}\;\mathrm{parasitemia}(\%)\\ =\frac{Total\;number\;of\;parasitized\;RBCs}{Total\;number\;of\;RCBs}\times 100\% \end{array}$$

#### 2.5.2 Organ index measurement

After blood collection, cervical vertebrae were removed, and the mice were sacrificed. The abdominal cavity was then opened. The spleens and livers of the mice were harvested and weighed, and the spleen and liver indices were calculated.


$$\begin{array}{c} \mathrm{Spleen}/\mathrm{Liver}\;\mathrm{index}\;(\%)\\ =\frac{Wet\;weight\;of\;spleen/liver}{Body\;weight}\times 100\% \end{array}$$

#### 2.5.3 Histopathological examination of spleen

The spleen was washed with physiological saline (0.9%), the excess liquid was absorbed using filter paper, and the tissue was immersed in 4% formaldehyde for fixation. Subsequently, it was dehydrated using an ethanol gradient (50, 70, 95, and 100% ethanol) and embedded in paraffin (n = 10 per group). The paraffin blocks were then sectioned at 3.5-μm thickness, stained with hematoxylin and eosin (H&E), and histological changes in the spleen were analyzed.

### 2.6 4D-data independent acquisition relative quantitative proteomics

#### 2.6.1 Extraction of total protein from mouse spleen tissue

The mice were sacrificed on D6, the abdominal cavities were opened, and the spleens were removed. The removed spleens were washed with physiological saline (0.9%). After the water from the spleen was absorbed using filter paper, the spleen was placed in a sterile RNase-free tube, frozen in liquid nitrogen, and stored at -80°C. For total protein extraction, frozen spleen tissue samples were taken out and transferred to a shaking tube. An appropriate amount of protein lysis solution (8 M urea + 1% SDS, containing protease inhibitors) was added. The tubes containing the samples were shaken using a high-throughput tissue grinder and lysed on ice. The supernatant was aspirated after centrifugation. The total protein content of each sample was determined using the bicinchoninic acid (BCA) method (Pierce™ BCA Protein Assay Kit; cat. no.: 23225; Thermo Fisher, USA). The samples were placed on ice throughout this experiment.

#### 2.6.2 Protein enzymolysis

To the spleen tissue protein sample (100 μg), lysis solution and ethylammonium bicarbonate buffer (100 mM) were added. Tris(2-carboxyethyl)phosphine (TCEP; 10mM) was added, and the mixture was incubated at 37°C for 60 min. Iodoacetamide (40mM) was added, and the solution was incubated at room temperature in dark. Acetone was added at 4°C, and the solution was incubated at -20°C for 4 h and centrifuged. Tetraethylammonium bromide (TEAB) was added to dissolve the precipitate. Subsequently, trypsin (cat. no.: 000053198; Promega, China) was added to the precipitate; the reaction conditions were: incubation at 37°C and enzymatic hydrolysis for 8 h. After trypsin digestion, the peptide fragments were dried using a vacuum pump. Following trypsin digestion, the peptides were dried using a vacuum pump. The dried peptides were then reconstituted with 0.1% trifluoroacetic acid (TFA). Desalting of the peptides was performed using an HLB cartridge, after which the samples were dried again in a vacuum concentrator. Peptide quantification was performed using Thermo Fisher Scientific Peptide Quantification Kit.

#### 2.6.3 DIA mass detection

Based on peptide quantification results, the peptides were analyzed by an Evosep One(Evosep)coupled with a tims TOF Pro2 mass spectrometer (Bruker, German) at Majorbio Bio-Pharm Technology Co. Ltd. (Shanghai, China). Briefly, the C18 column (150 μm×15cm, Evosep) was used with solvent A (water with 0.1% formic acid) and solvent B (ACN with 0.1% formic acid). The peptides were eluted using the 30 SPD gradient at the the flow rate of 500 nL/min.

Data-independent acquisition (DIA) data were acquired using a tims TOF Pro2 mass spectrometer operated in DIA-PASEF mode. MS data were collected over an m/z range of 400 to 1200 and an ion mobility range of 0.57 to 1.47 Vs·cm^−2^. Both accumulation time and ramp time were set to 100 ms. During MS/MS data collection, each tims cycle contained one MS and ten PASEF MS/MS scan. Exclusion was active after 0.4 min. A total of 64 DIA-PASEF windows were used (25 Th isolation windows).

#### 2.6.4 DIA data analysis

A digital spectrum library was established using ProteomeDiscoverer™ software version 2.4. After ion peak extraction from the raw DIA data, the spectrum library was imported into Spectraut™. Using iRT, the retention times were corrected, and six peptide segments and three daughter ions were selected. The quantitative analysis conditions were: protein ≤ 0.01; peptide segment ≤ 0.01; peptide confidence ≥ 99%; extracted ion chromatogram (XIC) width ≤ 75 ppm. The next step was to exclude peptide segments and calculate the peak areas.

#### 2.6.5 Bioinformatic analysis

The thresholds of fold change (> 1.2 or< 0.83) and *P*-value< 0.05 were used to identify differentially expressed proteins (DEPs). All identified proteins were annotated using the GO (http://geneontology.org/) and KEGG (http://www.genome.jp/kegg/) pathways. The DEPs were further used for GO and KEGG enrichment analyses. Protein–protein interaction analysis was performed using String version 10.5.

### 2.7 Western blotting

Mice spleen tissues (n = 3) were weighed. Radioimmunoprecipitation assay (RIPA) lysate (cat. no.: R0020; Solarbio, China; 10 μl/mg tissue), protease inhibitor, and phosphatase inhibitor were added at a ratio of 100:1:1, and the tissue samples were frozen at -80°C overnight. The tissue was homogenized, and the total protein content of each sample was determined using the BCA method. The volume of each sample was adjusted to achieve equal protein concentrations across all samples. The proteins were denatured by boiling the samples in a water bath for 5 min, and SDS-PAGE was performed. A constant current of 200 mA was applied to the flow membranes (polyvinylidene fluoride [PVDF] membranes; Millipore, USA) for 30 min.

Subsequently, the membranes were blocked with 5% skim milk for 1 h at room temperature. Subsequently, they were incubated in the primary antibody solution overnight at 4°C. The PVDF membranes were then incubated with secondary antibodies (cat. no.: S2001; Simubiotech, China; cat. no.: AS003; Abclonal, China) for 2 h. Finally, protein expression was analyzed using an ECL reagent (cat. no.: G2014; Servicebio, China). The antibodies used were as follows: SRC (cat. no.: 2108S); phosphoinositide 3-kinase (PI3 kinase) p85α (6G10); mouse mAb (cat. no.: 13666S); phospholipase C gamma 2 (PLCγ2; cat. no.: 3872T); spleen tyrosine kinase (SYK; cat. no.: 2712T); phospho-myristoylated alanine-rich C-kinase substrate (MARCKS; Ser152/156; cat. no.: 2741S); cell division cycle 42 (CDC42; cat. no.: 2462S); RAC1/2/3 (cat. no.: 2465S); all these antibodies were procured from Cell Signaling Technology (USA). Anti-PKC was procured from Abcam, UK (cat. no.: ab181558).

### 2.8 Statistical analysis

All values are presented as mean ± standard error of the mean (SEM). GraphPad Prism (version 8.0) was used for statistical analyses. Differences among three or more groups were determined using one-way analysis of variance (ANOVA) or the Kruskal–Wallis test. Statistical significance was set at *P*< 0.05.

## 3 Results

### 3.1 Antimalarial combination ZF-CQ shows higher antimalarial activity than that of artemisinin alone

The four-day suppressive test results revealed that the artemisinin-based combination, ZF-CQ, showed a better antimalarial activity against artemisinin-resistant *P. berghei*-infected mice.

**Figure 2:**
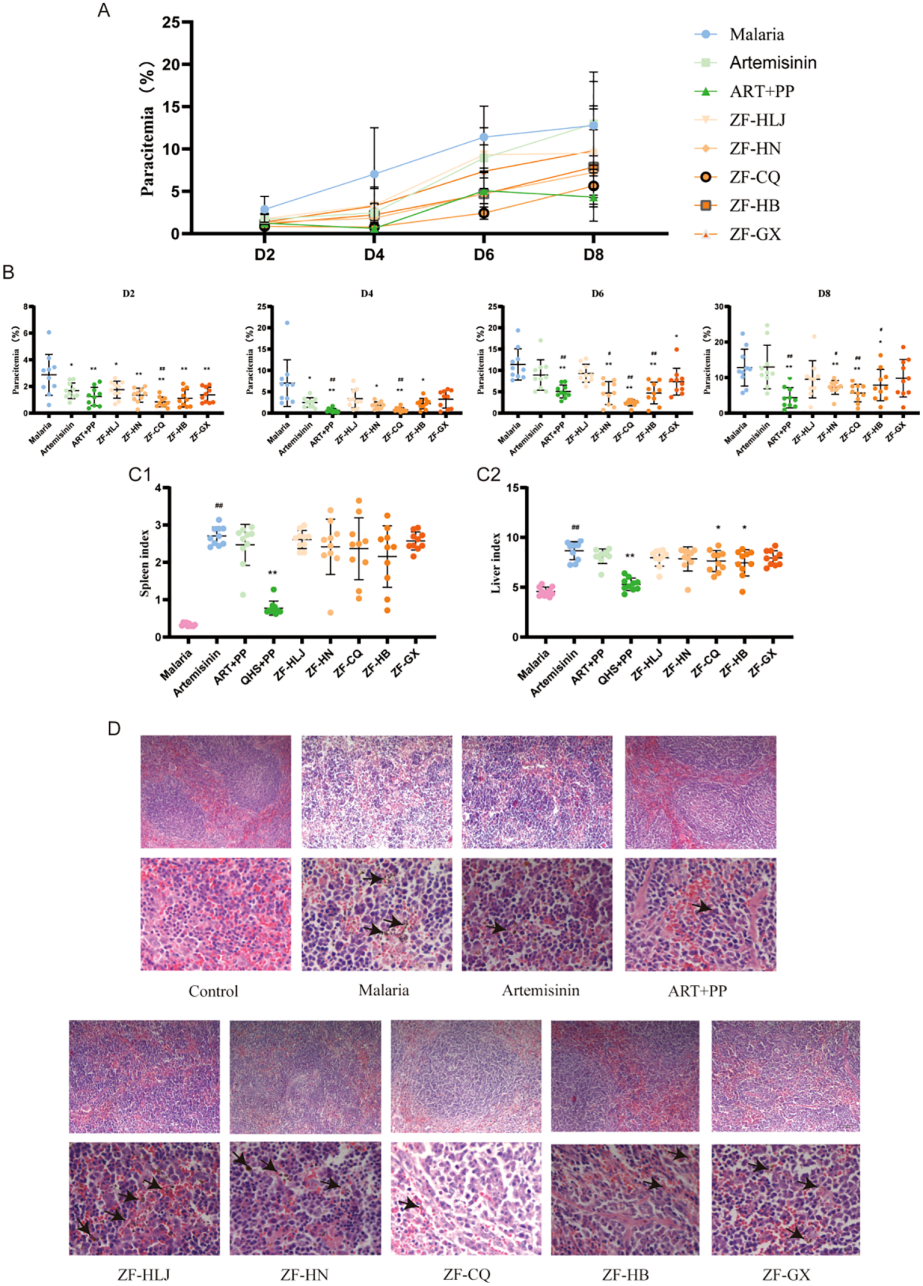
Therapeutic effects of different combinations of antimalarial compounds on mice with malaria (n = 10). **(A)** Growth curves of protozoa in different groups; **(B)** protozoal infection rates in different groups on D2, D4, D6 and D8; **(C1)** spleen indices of mice in different groups; **(C2)** liver indices of mice in different groups; **(D)** hematoxylin and eosin (H&E) staining of spleen tissues of mice in different groups (×100; ×400).

The four-day suppressive test results revealed that the protozoan infection rates of mice in the ART + PP, ZF-HN, ZF-CQ, and ZF-HB groups were significantly reduced on D2, D4, D6, and D8 (*P*< 0.01 or< 0.05) and those in the ZF-GX and ZF-HLJ groups were significantly reduced on D2 (*P*< 0.01 or< 0.05) compared with that in the malaria group. The protozoan infection rate of mice in the ZF-CQ group was significantly reduced on D2, D4, D6, and D8 (*P*< 0.01 or< 0.05), that in the ART + PP group was significantly reduced on D4, D6, and D8 (*P*< 0.01), and those in the ZF-HN and ZF-HB groups were significantly reduced on D6 and D8 compared with that in the artemisinin group. The protozoan infection rate in the ZF-CQ group was significantly lower than that in the ART + PP group (*P*< 0.01) (**Figures 2A, B**).

Analysis of the organ indices revealed that the spleen and liver indices of the malaria group were significantly higher (*P<* 0.01) compared with those of the control group; compared with that of the malaria group, the liver indices of ZF-CQ and ZF-HB groups were significantly lower (*P<* 0.05) (**Figures 2C1, C2**).

H&E staining of the spleens of different groups revealed that in the control group, the spleen capsule was complete and a large amount of white pulp could be seen. The white pulp was wrapped in the marginal zone, and the boundary between the red and white pulps was clearly visible. The lymphocytes in the white pulp were dense, the number of red pulp cells was low (mainly RBCs), the number of lymphocytes was low, and the cell morphology was regular. In the malaria group, the spleen capsule was thickened, the red pulp area of the spleen was expanded, the number of lymphocytes was high, the shape was irregular, the structure of the white pulp area was destroyed, the marginal area had disappeared, the spleen tissue appeared as a vacuole-like structure, and a significant degree of malarial pigmentation was observed. After administration of the drugs, the parenchymal structure of the mouse spleen recovered, and the marginal zone became clear; malaria pigmentation in the ZF-CQ and ZF-HB groups decreased significantly, and the spleen tissue structure was clear (**Figure 2D**).

### 3.2 4D-DIA relative quantitative proteomic identification and quantification

To comprehensively evaluate the changes in splenic proteins caused by ZF-CQ treatment, we performed a 4D-DIA relative quantitative proteomic experiment. Twelve samples were subjected to proteomic analysis, of which 1–3 comprised the control group, 4–6 comprised the malaria group, 7–9 comprised the artemisinin group, and 10–12 comprised the ZF-CQ group. Quality inspection was performed using SDS-PAGE. The results (**Supplementary Figure S1A**) showed that the sample bands were clear and rich, meeting the requirements for subsequent experiments.

Using the established database to search for raw mass spectrometry data offline, we obtained peptide number 38261 and the protein numbers 5267. The quality-controlled protein and peptide segments were counted to analyze the distribution of the number and length of the peptide segments and proteins (**Supplementary Figures S1B–D**). Principal component analysis (PCA) revealed that samples from the same group clustered well, whereas those from different groups were markedly separated (**Supplementary Figure S1E**). In order to show the correlation between samples, we randomly selected two samples, calculated the correlation coefficient based on their expression levels, and expressed the correlation between the two samples in the form of a heat map based on the correlation coefficient value. The results for samples from this test group were reproducible (**Supplementary Figure S1F**). These results indicated that the 4D-DIA proteomic data were of high quality and reliable.

### 3.3 Differentially expressed protein analysis

**Figure 3:**
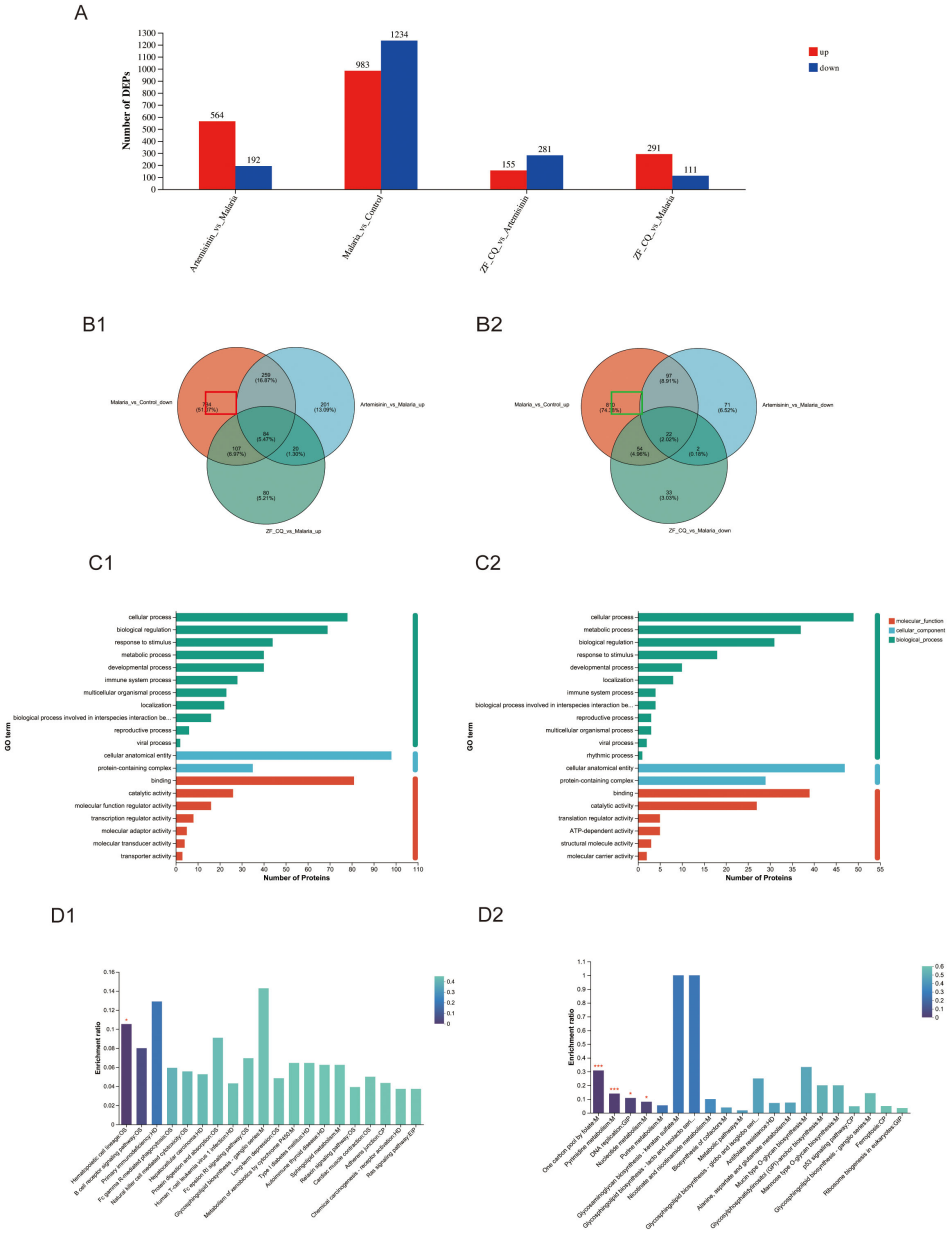
Proteomic analysis of mouse spleen tissue. **(A)** Statistical chart showing differences in protein expression levels; **(B)** Venn diagram for differential protein screening; specific upregulated (**B1**; red box) and downregulated (**B2**; green box) proteins; **(C1)** GO analysis of ZF-CQ-specific upregulated DEPs; **(C2)** GO analysis of ZF-CQ-specific downregulated DEPs; **(D1)** KEGG analysis of ZF-CQ-specific upregulated DEPs; **(D2)**: KEGG analysis of ZF-CQ-specific downregulated DEPs.

DEPs were screened based on the criteria of a 1.2-fold variation in expression levels and a *P*-value of< 0.05. We identified 2217 DEPs between the malaria and control groups (983 upregulated and 1234 downregulated), 756 DEPs between the artemisinin and malaria groups (564 upregulated and 192 downregulated), 402 DEPs between the ZF-CQ and malaria groups (291 upregulated and 111 downregulated), and 436 DEPs between the ZF-CQ and artemisinin groups (155 upregulated and 281 downregulated) (**Table 2**; **Figure 3A**).

**Table 2:** Differentially expressed proteins between different groups.

Different groups	Total proteins	Upregulated	Downregulated
Malaria vs. control	2217	983	1234
Artemisinin vs. malaria	756	564	192
ZF-CQ vs. malaria	402	291	111
ZF-CQ vs. artemisinin	436	155	281

### 3.4 Screening of specific DEPs in ZF-CQ group

Three protein sets (Malaria_vs_Control_down, Artemisinin_vs_Malaria_up, and ZF_CQ_vs_Malaria_up) were subjected to Venn analyses. By examining the overlapping relationships among the protein sets, we identified the intersection between the complements of Malaria_vs_Control_down relative to Artemisinin_vs_Malaria_up and ZF_CQ_vs_Malaria_up sets (indicated by the red box in **Figure 3B1**). This subset represented 107 specifically upregulated DEPs in the ZF-CQ group compared to those in the artemisinin monotherapy group in the splenic tissues of murine malaria models (total, 107).

Similarly, Venn analysis of three protein sets (Malaria_vs_Control_up, Artemisinin_vs_Malaria_down, and ZF_CQ_vs_Malaria_down) revealed an intersection between the complement of Malaria_vs_Control_up relative to the Artemisinin_vs_Malaria_down and ZF_CQ_vs_Malaria_down sets (marked by the green box in **Figure 3B2**). This cohort comprised 54 specifically downregulated DEPs in the ZF-CQ group compared to those in the artemisinin group in the same model system (total, 54).

### 3.5 Functional analyses of differentially expressed proteins in ZF-CQ group

Gene Ontology (GO) analysis of the 107 ZF-CQ-specific upregulated DEPs revealed predominant enrichment of cellular anatomical entities and protein-containing complexes under cellular components. Molecular functions were dominated by binding and catalytic, molecular function regulatory, and transcriptional regulatory activities. These DEPs participated in 16 biological processes, including cellular processes, biological regulation, responses to stimuli, metabolic processes, and developmental processes (**Figure 3C1**).

KEGG analysis indicated that the upregulated common DEPs were primarily involved in cancer pathways, metabolic pathways, focal adhesion, B cell receptor signaling pathway, hematopoietic cell lineage, human T-cell leukemia virus 1 infection, Epstein-Barr virus infection, Fc gamma R-mediated phagocytosis, and PI3K-AKT signaling pathway (**Figure 3D1**).

Gene Ontology (GO) analysis of the 54 ZF-CQ-specific downregulated DEPs revealed predominant enrichment in cellular anatomical entities and protein-containing complexes under cellular components. The molecular functions were dominated by binding and catalytic, transcriptional regulatory, and ATP-dependent activities. These DEPs participated in 14 biological processes, including cellular, metabolic, and biological regulation, responses to stimuli, and developmental processes (**Figure 3C2**). KEGG analysis indicated that the common downregulated DEPs were primarily involved in metabolic pathways, pyrimidine metabolism, nucleotide metabolism, and cofactor biosynthesis (**Figure 3D2**).

### 3.6 Protein-protein interactions and core DEP screening

A total of 54 DEPs were downregulated in the ZF-CQ group, of which 33 DEPs were related. The functions of these 33 DEPs were analyzed, PPIs were mapped, and 17 core DEPs were identified as follows: minichromosome maintenance complex 7 (MCM7), ubiquitin-like with PHD and RING finger domains 1 (UHRF1), periodic tryptophan protein 1 (PWP1), methylenetetrahydrofolate dehydrogenase/methenyltetrahydrofolate cyclohydrolase 2 (MTHFD2), carbamoyl-phosphate synthetase 2, aspartate transcarbamylase, and dihydroorotase (CAD), MTHFD1, FCF1, serine hydroxymethyltransferase 2 (SHMT2), thymidylate synthetase (TYMS), WDR75, PRIM2, cytidine deaminase (CDA), ribonucleotide reductase M1 (RRM1), WDR36, MCM3, replication factor C5 (RFC5), and notchless 1 (NLE1). Analysis of the KEGG pathways associated with the core downregulated DEPs showed that most DEPs were concentrated in pathways related to the cell cycle, such as amino acid metabolism, nucleotide metabolism, and DNA replication (**Supplementary Figure S2A**).

A total of 107 DEPs were upregulated in the ZF-CQ group, of which 32 DEPs were related. The functions of these 32 DEPs were analyzed, PPIs were mapped, and 14 core DEPs were identified: H2-DMa, Serpinb1a, CR2, CD5, CD2, Wiskott-Aldrich syndrome protein (WAS), PSTPIP1, GRB2-related adapter protein 2 (GRAP2), hematopoietic cell-specific Lyn substrate 1 (HCLS1), vasodilator-stimulated phosphoprotein (VASP), LASP1, FYB1, tensin 3 (TNS3), and SH3PXD2A. KEGG pathway analysis of core downregulated DEPs showed that most DEPs were concentrated in pathways related to B cell receptor signaling, hematopoietic cell lineage, and Fc gamma R-mediated phagocytosis (**Supplementary Figure S2B**).

### 3.7 Study of mechanism and validation of key proteins associated with ZF-CQ

We aimed to investigate the potential mechanism of action of ZF-CQ against malaria. We concentrated on the ZF-CQ-specific upregulated DEPs, which were enriched in different KEGG pathways. The DEPs were sorted according to the numbers of proteins enriched in the associated pathways. The top few pathways were hematopoietic cell lineage (four DEPs), Fc gamma R-mediated phagocytosis (three DEPs), chemokine signaling pathway (three DEPs), and focal adhesion (three DEPs).

**Figure 4:**
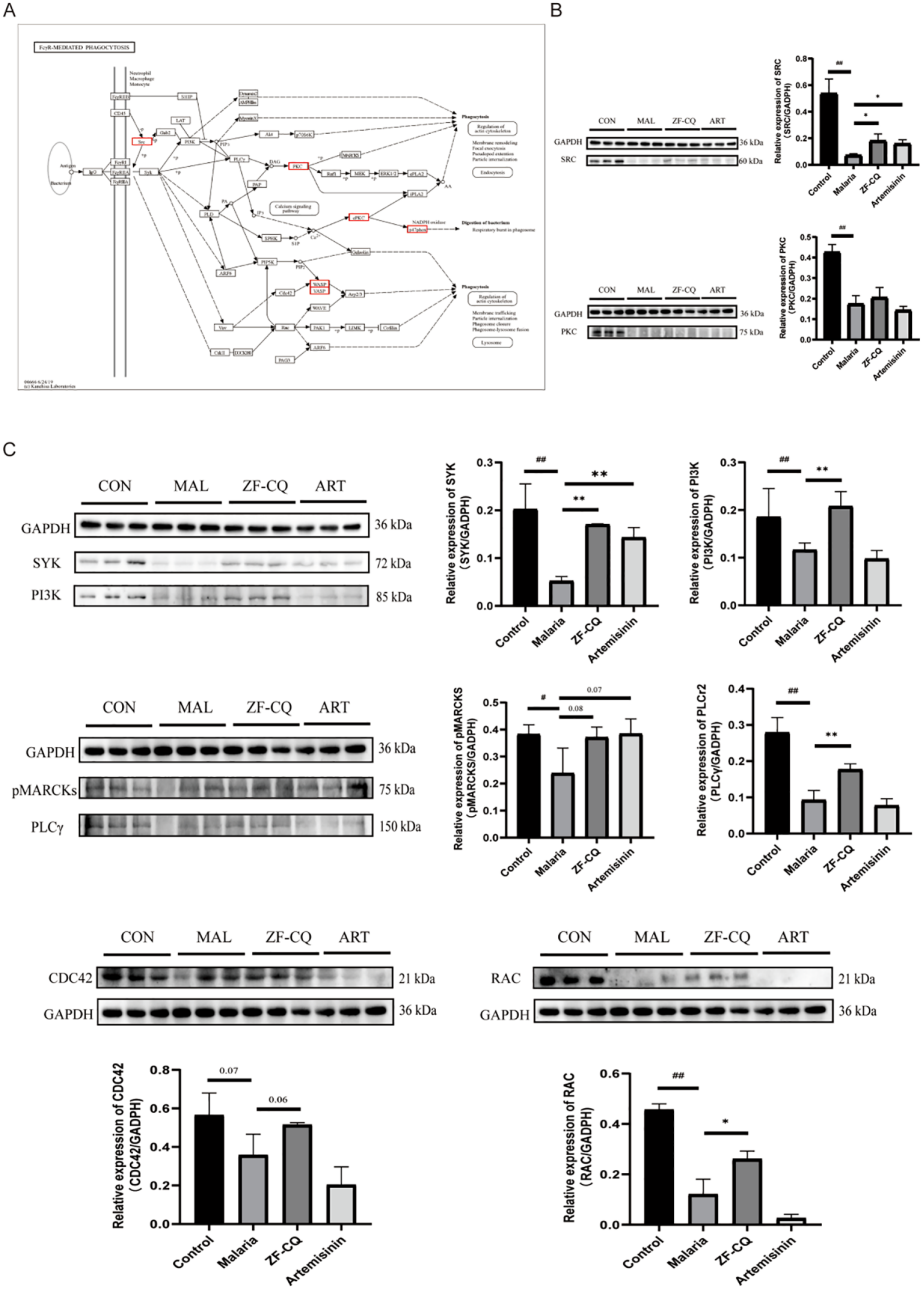
Results of western blot (WB) analysis of Fc gamma R (FcγR)-mediated phagocytosis and related proteins. **(A)** FcγR-mediated phagocytosis and core differentially expressed proteins (red box); **(B)** WB imaging and relative expression levels of major differentially expressed proteins SRC and PKC; **(C)** WB imaging and relative expression levels of key proteins for FcγR-mediated phagocytosis.

We validated the results of proteomic analysis and elucidated the molecular mechanisms underlying the role of ZF-CQ in malaria pathogenesis. Western blotting results (**Figure 4**) indicated that the expression levels of proteins SRC and PKC in the spleens of the malaria group decreased significantly (*P<* 0.01 or< 0.05, respectively) compared with those in the control group. Compared with that in the malaria group, the expression level of protein SRC in the spleen of the ZF-CQ group was significantly higher (*P<* 0.05).

Moreover, the Fc gamma R-mediated phagocytic pathway is one of the most direct and effective immune clearance methods. As shown in **Figure 4**, compared with those in the control group, the expression levels of key node proteins of Fcγ R-mediated phagocytosis, including SYK, PI3K, PLCγ2, p-MARCKS, CDC42, and RAC, in the spleen samples of the malaria group decreased significantly (*P<* 0.01 or< 0.05). Compared with those in the malaria group, the expression levels of proteins SYK, PI3K, PLCγ2, and RAC in the spleens of mice in the ZF-CQ group increased significantly (*P<* 0.01 or< 0.05), and the expression levels of proteins p-MARCKS and CDC42 showed an upward trend. These findings indicated that ZF-CQ inhibited Fc gamma R-mediated phagocytosis.

## 4 Discussion

Since the discovery of artemisinin, nearly 200 compounds have been isolated from *A. annua (*17). The major compounds include terpenoids, coumarins, volatile oils, and flavonoids. In addition to artemisinin, other compounds present in *A. annua*, such as arteannuin B, artemisinic acid (15), and scopoletin, have good pharmacological activities, including antipyretic, immune improvement, and antimalarial activities (18). The combination of artemisinin with arteannuin B, artemisinic acid, and scopoletin has shown antimalarial activity (10, 12). In this study artemisinin remains the principal component responsible for parasiticidal activity. Arteannuin B, artemisinic acid, and scopoletin do not have antimalarial activity when used alone, as previously confirmed by Tu Youyou’s studies in mice and our research group’s *in vitro* experiments with human *Plasmodium falciparum* (**Supplementary Figure S3**).

The reasons for these results, on one hand, may be due to the enzymatic interaction of arteannuin B, arteannuic acid, and scopoletin with artemisinin, which can inhibit the metabolic enzymes. On the other hand, these three components may affect the binding of artemisinin with serum albumin, resulting in changes in protein storage and transport of artemisinin *in vivo*. Preliminary studies showed that the quenching constant and binding constants of artemisinin with bovine serum albumin in the four-component combination group increased compared to the single artemisinin group, which can affect the pharmacokinetic behavior of artemisinin (19).

In this study, the five groups set up based on component ratio (ZF-HLJ, ZF-HN, ZF-CQ, ZF-HB, and ZF-GX) screening correspond to the contents of artemisinin, arteannuin B, artemisinin acid, and scopoletin in *Artemisia annua* L. produced in five different provinces of China (18, 20). When developing new ACTs, it is important to explain the *Dao-di* herbs theory of traditional Chinese medicinal materials from a certain perspective (21).

The spleen, the body’s largest secondary lymphoid organ, plays a pivotal role in maintaining circulatory homeostasis through specialized filtration mechanisms and immune surveillance capabilities. Resident macrophages selectively sequester and eliminate senescent or parasitized erythrocytes, thereby ensuring blood flow integrity and pathogen clearance (22). However, during *Plasmodium* infection, this finely tuned system undergoes pathological hyperactivation, leading to splenic enlargement (splenomegaly) due to excessive immune cell recruitment and tissue remodeling. Clinical comparisons between patients with malaria who have been splenectomized and whose spleens are intact revealed stark differences in parasite clearance kinetics. Intact spleens enable complete parasite elimination within seven days of artemisinin-based therapy, whereas individuals who have undergone splenectomy require 8–9 weeks post-treatment to achieve a comparable efficacy of therapy. These observations underscore the indispensable role of the spleen as a frontline defense organ against blood-stage parasites (23). Pharmacological studies have demonstrated that artemisinin derivatives synergize splenic filtration and monocyte-mediated phagocytosis, amplifying oxidative stress pathways and accelerating the removal of parasitized erythrocytes. Such mechanistic insights have redirected research toward deciphering splenic immunobiology, positioning it as a critical target for host-directed therapeutic strategies against drug-resistant *Plasmodium* species (24).

The spleen is responsible for clearing foreign bodies and aging or damaged RBCs; it also triggers an immune response to blood-borne antigens (25, 26). A specific function of the spleen is to recognize changes in the size, shape, and ability to deform in macrophages (MPs), which are highly sensitive to these changes. When the flow rate of blood slows down after entering RBCs, the blood first comes into contact with immune cells, such as MPs and dendritic cells (DCs). MPs recognize and ingest pathogens, cell debris, and aging RBCs. Some pathogens can be directly recognized by MPs; however, many pathogens require “special treatment.” In this process, the surface of the pathogen is wrapped with other substances such as complements or tryptophan. These substances interact with MP receptors and promote pathogen clearance. Trapped pathogens or cell debris are recognized and cleared by red pulp macrophages (27). Compared with MPs derived from blood monocytes, red pulp giant cells mainly express the low-affinity receptor FCγRIA; FCγRIIA does not express the inhibitory FCγRIIB receptor but expresses very low levels of the high-affinity receptor FCγRI (28).

Based on the results of proteomic analysis, we focused this study on FcγR-mediated phagocytosis. In order to verify the results of proteomic analysis and elucidate the regulation of FcγR-mediated phagocytosis by ZF-CQ, we conducted western blot analysis of the expression levels of candidate proteins SRC and PKC and key node proteins SYK, PI3K, PLCγ, p-MARCKS, CDC42, and RAC during FcγR-mediated phagocytosis. The results showed that the antimalarial formula ZF-CQ promoted the expression of the detected proteins.

Phagocytosis and clearance are initiated by the mutual recognition of phagocytic receptors and surface molecules of phagocytic particles by monocyte macrophages (29). It plays a decisive role in various functions, such as the phagocytosis of large particles and clearance of apoptotic cells in eukaryotes, as well as in other host defense mechanisms. FcγR-mediated phagocytosis is one of the most direct and effective immune clearance methods of the body’s immune system (30). During the immune response, when IgG recognizes and binds to a specific antigen, it activates FcγR, which phagocytes and digests pathogens, cellular garbage, and tumor cells by regulating the phagocytic behavior of MPs, DCs, and NK cells. This phagocytosis is effective, because disease-related substances are cleared quickly, protecting the body from harm. Subsequently, FcγR-mediated stimulating signals enter cells, and downstream signal proteins such as PI3K, PKC, and GTPase Rho family (such as Rho, RAC, and CDC42) undergo changes, which lead to actin polymerization and phagosome formation (31).

PI3K is a cytoplasmic complex that catalyzes the phosphorylation of D-3 phosphatidylinositol (32, 33). Crosslinking of FcγRI and FcγRII increases the activity of PI3K, and inhibition of PI3K blocks FcγR-mediated phagocytosis (34, 35). PI3K can affect the aggregation of (1) integrins and actinins and regulate cell shape; therefore, PI3K is necessary for FcR- and CR3-mediated phagocytosis and may be an important mediator in the signaling cascade that induces phagocytosis (36). The tyrosine phosphorylation cascade of FcγR begins with a member of the SRC family of protein tyrosine kinases. In addition, the process of FcγR-mediated polymerization of phagocytic vesicular actin in MPs from SYK-free mice was similar to that observed in normal mouse MPs but could not be fully internalized. All of the above studies suggested that PI3K participates in FcγR-mediated phagocytosis and relies on the SYK signal transduction pathway (37).

Protein kinase C (PKC) has an important role in phagocytosis, and different isoforms of PKC regulate different aspects of swallowing. Studies have shown that during the phagocytosis of giant cells mediated by FcγR, CR3, and mannose receptors, PKCα isoenzymes can quickly bind to primary phagosomes and persist until maturation (38). The two substrates involved in PKC endocytosis are MARCKS and MacMARCKS, which are actin filament crosslinking proteins that connect actin to cell membranes and may be involved in the regulation of actin remodeling during phagosome formation.

Different members of the Rho family, a factor downstream of FcγR-mediated phagocytosis, have different phagocytic effects (36). Recombinant cell division cycle protein 42 (CDC42) is involved in the formation of filamentary feet and activation of Ras-related C3 botulinum toxin substrate 1 (RAC); RAC stimulation leads to membrane wrinkling and Ras homology protein (Rho) activation, and Rho stimulation results in local adhesions and stress fiber formation (36).

In addition to their well-established antimalarial effects, recent studies have indicated that artemisinin drugs can influence the immune response by modulating the polarization state of macrophages. Research has demonstrated that artemisinin and its derivatives can inhibit the activation of lipopolysaccharide-induced pro-inflammatory macrophages (M1 phenotype) (39, 40). Regarding the regulation of red pulp macrophages in the spleen, artemisinin can promote the transition of macrophages towards the anti-inflammatory phenotype (M2) by inducing the expression of heme oxygenase-1 (HO-1). This polarization shift contributes to the maintenance of iron metabolism homeostasis within the red pulp (41).

The results of this study showed that the expression levels of key node proteins SYK, PI3K, PLCγ, p-MARCKS, CDC42, and RAC during FcγR-mediated phagocytosis in the spleens of mice in the ZF-CQ group were upregulated to varying degrees compared with those in the malaria group; the expression levels in the ZF-CQ group were higher than those in the artemisinin group. This suggests that the antimalarial components of *A. annua*, especially the non-artemisinin components, may enhance the anti-malarial efficacy of artemisinin by modulating FcγR-mediated phagocytosis in the host mice.

## 5 Conclusions

This study demonstrated that the ratio of artemisinin: arteannuin B: artemisinic acid: scopoletin = 80.9:15.8:2.0:1.3 shows the best antimalarial activity. The favorable liver/spleen indices indicated the advantage of combination over artemisinin monotheraphy. This formula regulates the phagocytic behavior of MPs, DCs, and NK cells through FcγR-mediated phagocytosis and enhances the body’s inhibitory effect on *Plasmodium*.

## Ethics approval

This study was approved by the Institutional Animal Care and Use Committee (IACUC) of the Institute of Chinese Materia Medica, China Academy of Chinese Medical Sciences (Beijing, China) under code number 2023B198.

## Data Availability

The original contributions presented in the study are included in the article and supplementary material, further inquiries can be directed to the corresponding authors.

## Supplementary material

Supplementary Figure 1 Proteomic quality control results. **(A)** Results of SDS-PAGE analysis of samples: 1–3 comprise the control group (Control), 4–6 comprise the model group (Malaria), 7–9 comprise the ART group (Artemisinin), and 10–12 comprise the ZF-CQ group (ZF_CQ); **(B)** distribution of numbers of peptide segments; **(C)** distribution of lengths of peptide segments; **(D)** protein coverage; **(E)** protein molecular weight distribution; **(F)** quantitative principal component analysis of all samples; **(G)** heat map showing correlation between samples.

Supplementary Figure 2 Protein–protein interaction networks. **(A)** Specific upregulated core protein network map. **(B)** Specific downregulated core protein network map.

Supplementary Figure 3 Median effective concentration (EC50) of artemisinin, arteannuin B, artemisinic acid, and scopoletin against human *P. falciparum.* In the legend, ART means artemisinin, ATS means artesunate, B: arteannuin B, C: artemisinic acid, D: scopoletin.
