# Supplementary material for: Proteomics-driven screening of artemisinin-based combination ratios and mechanistic insights into Plasmodium berghei infection in mice
Source: Front Immunol. 2025 Dec 5;16:1716096. doi: 10.3389/fimmu.2025.1716096 (PMC12715000; doi:10.3389/fimmu.2025.1716096)
Supplement: Supplementary Figure 1 — Proteomic quality control results. (A) Results of SDS-PAGE analysis of samples: 1–3 comprise the control group (Control), 4–6 comprise the model group (Malaria), 7–9 comprise the ART group (Artemisinin), and 10–12 comprise the ZF-CQ group (ZF_CQ); (B) distribution of numbers of peptide segments; (C) distribution of lengths of peptide segments; (D) protein coverage; (E) protein molecular weight distribution; (F) quantitative principal component analysis of all samples; (G) heat map showing correlation between samples. [file DataSheet1.doc]

**Supplementary material**


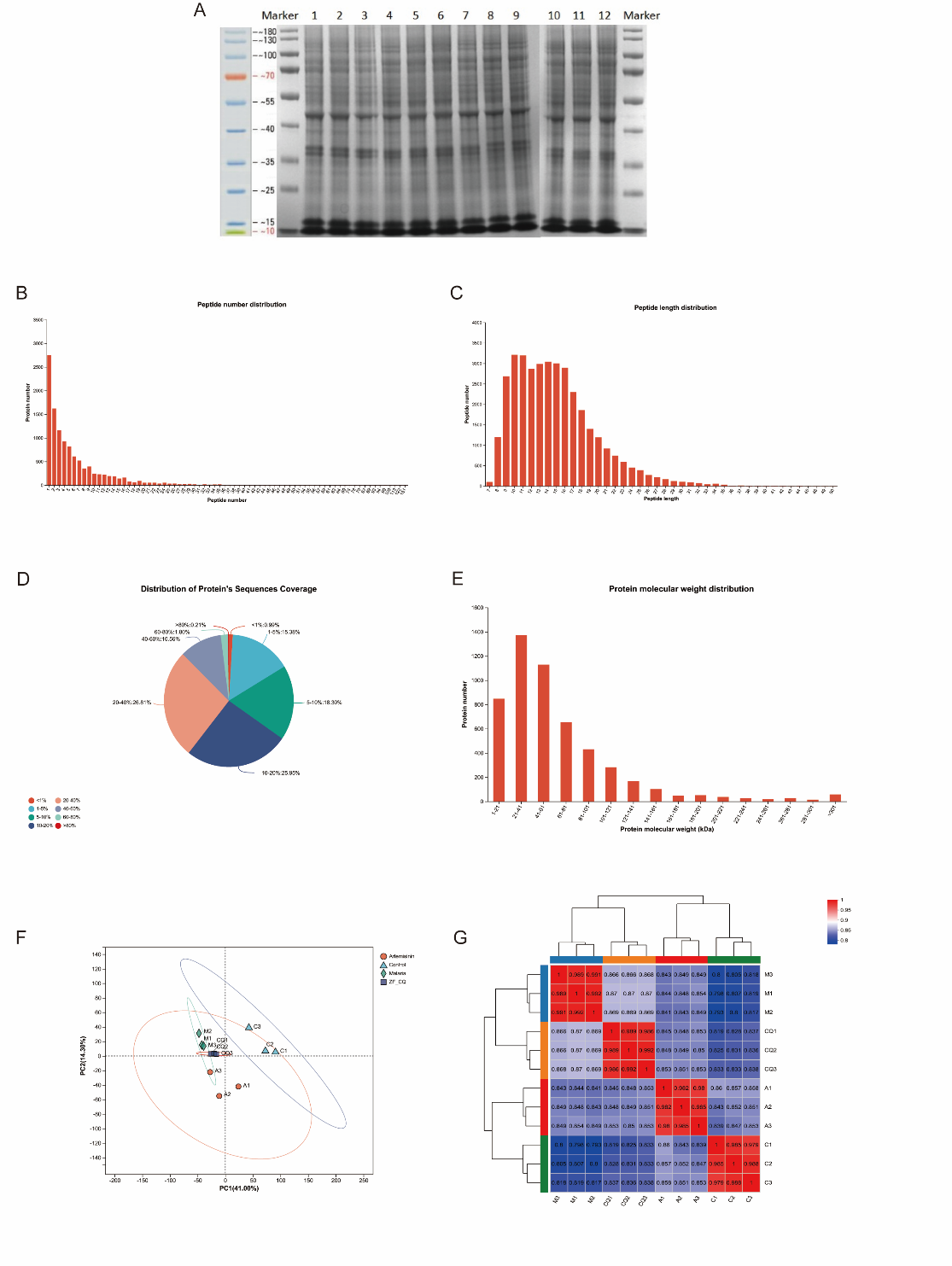


Fig. S1. Proteomic quality control results. (A) Results of SDS-PAGE analysis of samples: 1–3 comprise the control group (Control), 4–6 comprise the model group (Malaria), 7–9 comprise the QHS group (Artemisinin), and 10–12 comprise the ZF-CQ group (ZF_CQ); (B) distribution of numbers of peptide segments; (C) distribution of lengths of peptide segments; (D) protein coverage; (E): protein molecular weight distribution; (F): quantitative principal component analysis of all samples; (G) heat map showing correlation between samples.


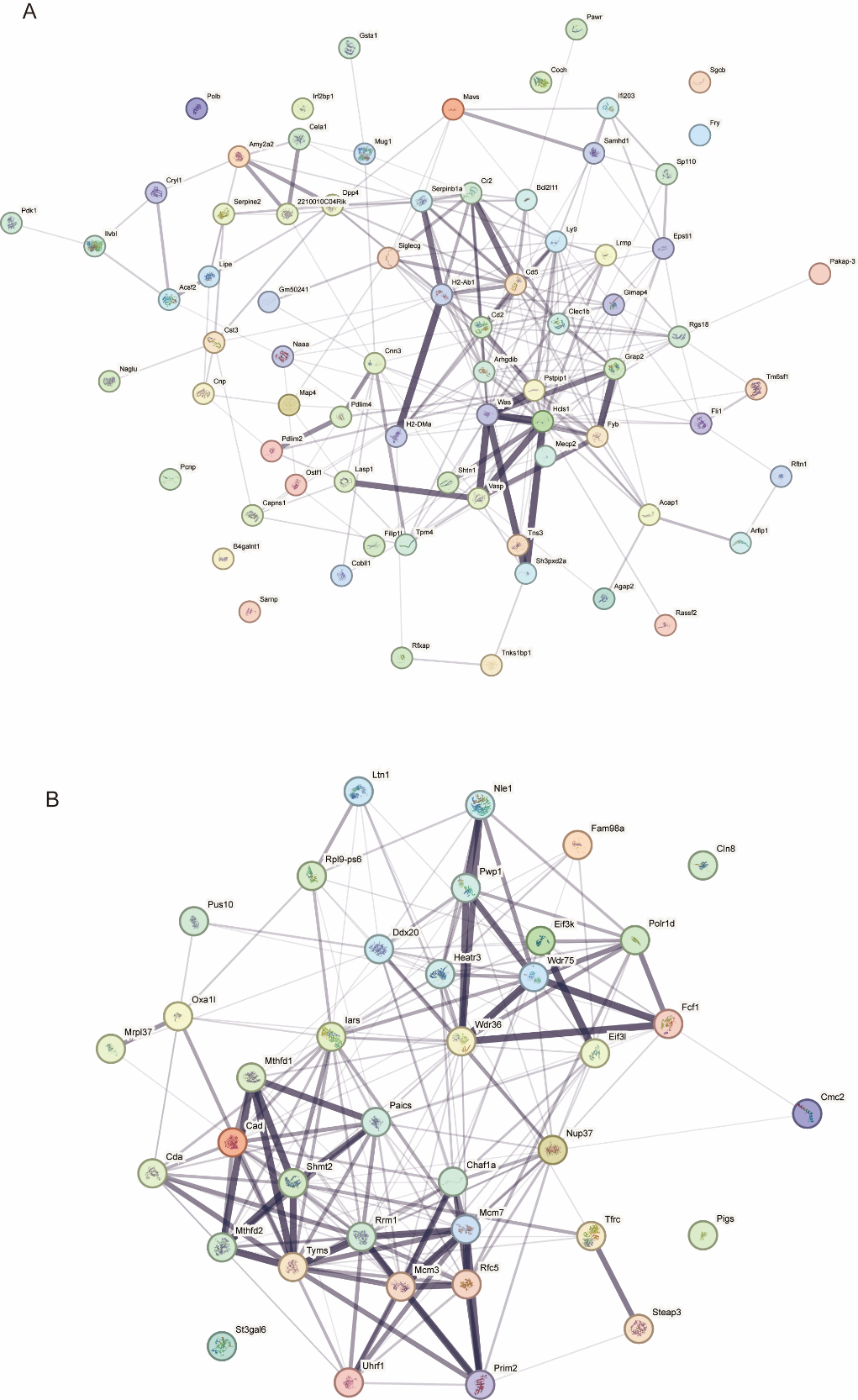


Fig. S2. Protein–protein interaction networks. (A) Specific upregulated core protein network map. (B) Specific downregulated core protein network map.


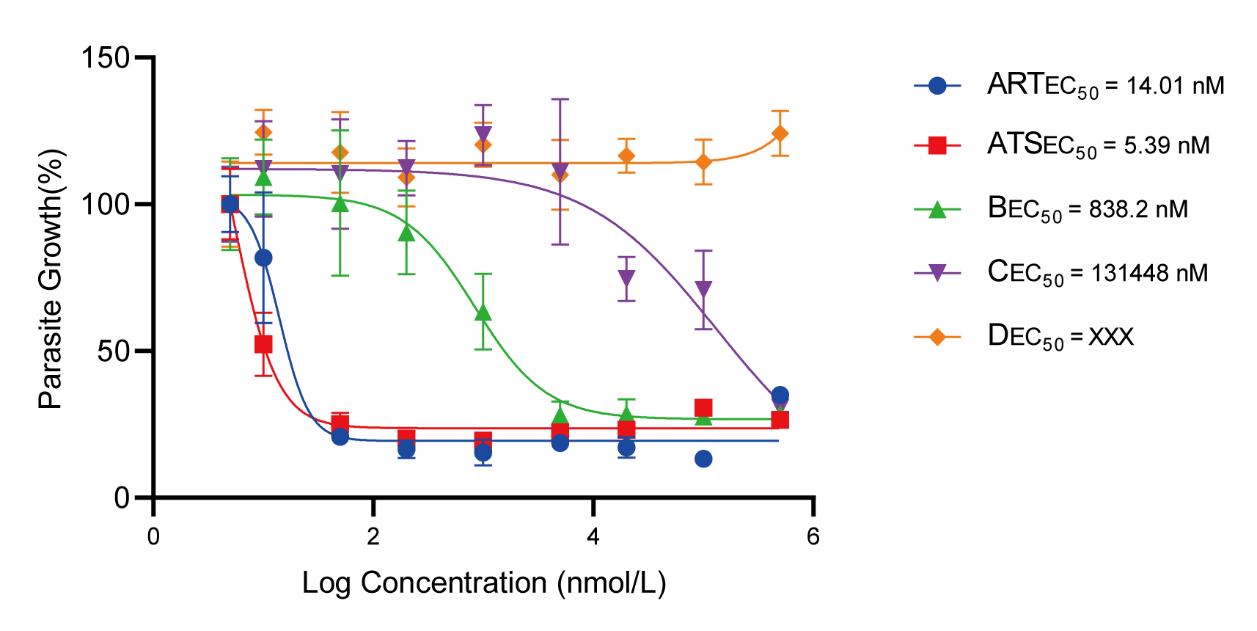


Fig. S3 Median effective concentration (EC50) of artemisinin , arteannuin B, artemisinic acid, and scopoletin against human *P. falciparum.* In the legend, ART means artemisinin, ATS means artesunate, B: arteannuin B, C: artemisinic acid, D: scopoletin.
